# Super-Resolution Imaging of Neuronal Structures with Structured Illumination Microscopy

**DOI:** 10.3390/bioengineering10091081

**Published:** 2023-09-13

**Authors:** Tristan C. Paul, Karl A. Johnson, Guy M. Hagen

**Affiliations:** UCCS BioFrontiers Center, University of Colorado Colorado Springs, 1420 Austin Bluffs Parkway, Colorado Springs, CO 80918, USA; tpaul@uccs.edu (T.C.P.); k9johnso@ucsd.edu (K.A.J.)

**Keywords:** fluorescence microscopy, structured illumination, brain, Bayesian methods, super-resolution

## Abstract

Super-resolution structured illumination microscopy (SR-SIM) is an optical fluorescence microscopy method which is suitable for imaging a wide variety of cells and tissues in biological and biomedical research. Typically, SIM methods use high spatial frequency illumination patterns generated by laser interference. This approach provides high resolution but is limited to thin samples such as cultured cells. Using a different strategy for processing raw data and coarser illumination patterns, we imaged through a 150-micrometer-thick coronal section of a mouse brain expressing GFP in a subset of neurons. The resolution reached 144 nm, an improvement of 1.7-fold beyond conventional widefield imaging.

## 1. Introduction

Recently developed methods for surpassing the diffraction limit in optical fluorescence microscopy include stimulated emission depletion microscopy (STED) [1], stochastic optical reconstruction microscopy (STORM) [2], photoactivated localization microscopy (PALM) [3], super-resolution optical fluctuation imaging (SOFI) [4], and structured illumination microscopy (SIM) [5,6]. These methods have had large impacts in many fields, with super-resolution microscopy previously being used in many applications, including imaging the a mouse brain using STED [7], STORM [8], and SIM approaches [9]. SIM methods have been used in many situations, including the rapid imaging of clinical samples [10].

SIM is a method in which sets of images are acquired with shifting illumination patterns. The subsequent processing of these image sets results in images with optical sectioning, resolutions beyond the diffraction limit (super-resolution), or both [5,6,11,12,13,14]. Since its emergence over two decades ago [15], SIM has matured as an imaging technique, with multiple proposed methods for generating the structured illumination patterns [12,13,14,15,16,17,18,19,20,21,22,23,24,25] and processing the image data [6,13,26,27,28,29,30]. Compared to other super-resolution techniques, the speed, high signal-to-noise ratio, and low excitation light intensities characteristic to SIM make it a good choice for imaging a variety of samples in three dimensions. As shown here, SIM is accomplished with a standard fluorescence microscope with some additional required elements, whereas other approaches such as light-sheet microscopy require more specialized setups. There is an increasing interest in the methods and applications of SIM, with the field seeing many recent (2022–2023) improvements [31,32,33,34,35,36,37], including new methods involving deep learning approaches [38,39].

The imaging method we used, maximum a posteriori probability SIM (MAP-SIM), uses a Bayesian framework to reconstruct super-resolution SIM images [26,29,40]. This method has advantages including flexibility in the range of SIM illumination patterns which can be used and, in our case, the ability to use patterns with lower spatial frequencies. This, in turn, allows imaging deeper into samples in which scattering degrades the high spatial frequency patterns which are more commonly used in SIM. When the SIM pattern is out of focus, it blurs rapidly with increasing depth, producing a high intensity of out of focus light. This results in reduced pattern contrast in the acquired images. Because of this, traditional super-resolution SIM methods are typically limited to an imaging depth of 10–20 μm [41,42]. Here, we used MAP-SIM to image a fixed, optically cleared, ~150-μm-thick mouse brain coronal slice expressing a neuronal GFP marker while achieving a lateral resolution of 144 nm.

To overcome the challenges of imaging deeper into brain tissues, SIM has previously been combined with two-photon excitation [16] or with adaptive optics for in vivo studies [43,44]. These methods have offered impressive results, but they do involve additional costs and require additional optical devices and expertise, reducing the number of labs that can use these approaches. Here, we used a simpler and more economical approach with a non-laser light source and open-source software for SIM [45].

## 2. Methods

The sample used for this work was an optically cleared, green fluorescent protein (GFP) -labeled coronal mouse brain slice. The slice was approximately 150 μm thick and was obtained from SunJin Lab (Hsinchu City, Taiwan). The supplier used a Thy1-GFP mouse strain, and they stated that the sample was prepared as follows:

1. cardiac perfusion with cold, freshly prepared 4% paraformaldehyde (PFA)
2. fixation of the dissected brain with a 4% PFA solution on an orbital shaker overnight at 4 °C followed by washing three times with phosphate-buffered saline (PBS) at room temperature
3. sectioning the brain manually using a vibratome followed by clearing of the slice with RapiClear 1.52 (SunJin Lab) overnight at room temperature
4. mounting of the cleared sample with fresh RapiClear 1.52 reagent in a 0.25-mm-deep iSpacer microchamber (SunJin Lab)

For the SIM imaging, we used a home-built set-up based on the same design as described previously [20,26,40,46]. The current SIM system was based on an IX83 microscope equipped with several objectives (Olympus, Tokyo, Japan). Illumination was provided by a liquid light guide-coupled Spectra-X light source (Lumencor, Beaverton, OR, USA) using the cyan channel, which had an emission maximum of 470 nm. The illumination was collimated by an achromatic 50 mm focal length lens (Thor labs, Newton, NJ, USA) and vertically polarized with a linear polarizer (Edmund Optics, Barrington, NJ, USA) before entering a polarized beam splitter (PBS) cube (Thor Labs) and reflecting onto a liquid-crystal on silicon (LOCS) microdisplay (Forth Dimension Displays, Dalgety Bay, Scotland, UK). This device is a ferroelectric reflective-type spatial light modulator. The pixels, which were turned on, rotated the polarization of the light by ~90 degrees, converting vertical polarization to horizontal polarization. The horizontally polarized output of the microdisplay then passed through the PBS and was imaged into the microscope using a 180 mm focal-length lens (SWTLU-C, Olympus). The emitted fluorescent light was filtered (using a GFP filter set with dichroic T495lpxr and ET525/50 emission filters; Chroma, Bellows Falls, VT, USA) and then imaged with an sCMOS camera (Zyla 4.2+, Andor). The illumination power density on the sample used a 100× objective that was measured at 2.542 W/cm^2^ without SIM patterning (widefield illumination) and at 0.214 W/cm^2^ with the SIM pattern active. Sample movements and focusing were controlled by an XY piezo Z stage (Applied Scientific Instrumentation, Eugene, OR, USA).

**Figure 1:**
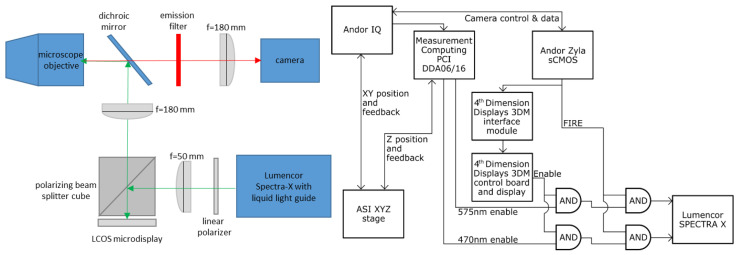
Simplified optical diagram (**left**) and connection diagram (**right**). The connection setup for the two-wavelength acquisition is shown, and in this study, only 470 nm illumination was used.

The microdisplay was used to produce the SIM patterns, and it was controlled by the software supplied with the device (MetroCon, Forth Dimension Displays). Various SIM patterns were used as shown in the supplementary material in Table S6. The pattern position was shifted by one pixel after each image was acquired such that the sum of all illumination masks resulted in homogenous illumination. Figure 1 shows a simplified diagram of the SIM optical system and a connection diagram illustrating how the microdisplay system was synchronized with the camera using IQ software (Andor) and a digital input/output computer card (DDA06/16, Measurement Computing, Concord, NH, USA). More details about the SIM system are given in the supplementary material. Table S1 shows a list of the components we used along with the manufacturer, part number, and vendor website. Tables S2–S5 show some of the relevant optical and performance characteristics of the camera, microdisplay, and light source. These details should be useful for those wishing to build their own SIM systems of this type. The supplementary text explains, and Figure S7 shows, a schematic of the timing scheme used by the SIM system, and they illustrate the function of the AND gates shown in Figure 1. The supplementary text also explains, and Figure S8 shows, additional details about the operation of the microdisplay.

## 3. Data Analysis

### 3.1. Optical Sectioning SIM (OS-SIM)

Several data processing methods are possible for generating optically sectioned images from SIM data (OS-SIM) [20,47]. The most commonly used implementation of this technique was introduced in 1997 by Neil et al. [15]. Their method worked by projecting a line illumination pattern onto a sample, followed by the acquisition of a set of three images with the pattern shifted by the relative spatial phases 0, 2π/3, and 4π/3, respectively. Using this method, an optically sectioned image can be recovered computationally as follows:

(1) $$I_{OS-SIM}={\left[{\left(I_{1}-I_{2}\right)}^{2}+{\left(I_{1}-I_{3}\right)}^{2}+{\left(I_{2}-I_{3}\right)}^{2}\right]}^{1/2},$$

where *I_OS-SIM_* is an optically sectioned image and *I*_1_, *I*_2_, and *I*_3_ are the three images acquired with the different pattern positions. This type of optically sectioned image is expected to be similar to that obtained with a laser scanning confocal microscope. If the sum of the individual SIM patterns results in homogeneous illumination, as was the case in our setup, a widefield (WF) image can also be recovered from the SIM data by taking the average of all images *I_n_*, as follows:

(2) $$I_{WF}=\frac{1}{N}\sum_{n=1}^{N}I_{n}.$$

This was the approach we used throughout this study to generate conventional widefield images.

Instead of using Equation (1), in this study, we used a method originally shown by Neil et al. [15] and later elaborated upon [20,47], as follows:

(3) $$I_{OS-SIM}=\left|\sum_{n=1}^{N}I_{n}\exp\left(2\pi i\frac{n}{N}\right)\right|.$$

We found that this method provided consistent results and could be applied when using any number of patterns instead of the three patterns used in the original work. The actual positions of the illumination patterns in the camera images were determined using a calibrated camera according to our previous work [15], and this was based on a well-known method for the spatial calibration of a camera [48].

### 3.2. SIM with Maximum a Posteriori Probability Estimation

MAP-SIM has been described previously [26]. In our study, the imaging process could be denoted as follows:

(4) $$y_{k}=HM_{k}x+n_{k},$$

where *M_k_* is a matrix in which the elements represent the *k*-th illumination pattern; ***y****_k_* denotes a low-resolution image acquired using the *k*-th illumination pattern; ***x*** is an unknown, high-resolution image; and ***n****_k_* is (Gaussian) additive noise. *H* is a matrix that models the convolution between the high-resolution image and the point-spread function (PSF) of the system. Each SIM image acquired generates an Equation (4) with a different illumination pattern (*k*). The linear system of Equation (4) produced in a SIM experiment must be solved in order to reconstruct a high-resolution image. This reconstruction can be defined as the inversion of the system of equations. In the presence of noise (***n****_k_*), the inversion becomes unstable and is considered an ill-posed problem. This means we need to add a constraint which stabilizes the inversion of the system and ensures the uniqueness of the solution. In this imaging model, the low-resolution images (***y****_k_*), high-resolution image (***x***), and nose (***n****_k_*) are measurement-dependent.

We modeled the PSF as an Airy disk which, in Fourier space, would lead to an optical transfer function (OTF) of the form [49] as follows:

(5) $$OTF\left(f\right)=\frac{1}{\pi }\left[2\cos^{-1}\left(\frac{f}{f_{c}}\right)-\sin\left(2\cos^{-1}\left(\frac{f}{f_{c}}\right)\right)\right],$$

where *f* is the spatial frequency. We estimated the cut-off frequency (*f_c_*) by calculating the radial average of the power spectral density (PSD) of a widefield image of 100 nm fluorescent beads [50]. This could also be calculated by taking the Rayleigh limit of the resolution *d* = 0.61λ/NA and expressing this value in terms of spatial frequency (1/*d*).

Using a Bayesian approach [26,27,29,51,52,53,54], high-resolution image estimation can be expressed as a minimized cost function according to the following:

(6) $$x_{\mathrm{HR}-\mathrm{MAP}}=\underset{x}{\arg\min}\left[\overset{K}{\underset{k=1}{\sum }}{\left\|y_{k}-HM_{k}x\right\|}^{2}+\lambda \Gamma \left(x\right)\right].$$

The cost function in Equation (6) consists of two terms. The first term describes the mean square error between the estimated HR image and the observed LR images. The second term (λΓ(***x***)) is a regularization term. To ensure positivity and promote a smoothness condition, we relied on quadratic regularization [54]. The contribution of Γ(***x***) was controlled by the parameter λ, a small positive constant defining the strength of the regularization (typically, λ = 0.01). We solved Equation (6) using gradient descent methods [54].

### 3.3. Spectral Merging

MAP estimation of high-resolution images obtained with structured illumination enables the reconstruction of high-resolution images (HR-MAP) with details that are unresolvable in a widefield microscope. However, MAP estimation, as described above, does not suppress out-of-focus light. On the other hand, the processing method according to Equation (3) used in optical sectioning SIM [15,20] provides images (LR-HOM) with optical sectioning. Noting that the unwanted, out-of-focus light was dominant at low spatial frequencies, we merged the LR-HOM and HR-MAP images in the frequency domain to obtain the final HR image (MAP-SIM). For 3D data, this is completed in a slice-by-slice fashion, resulting in a Z-stack of SIM images. Frequency-domain Gaussian low-pass filtering was applied to the LR-HOM image, and a complementary high-pass filter was applied to the HR-MAP image. We used a weighting scheme that could be described by the following equation:

(7) $$x_{\mathrm{MAP}-\mathrm{SIM}}=F^{-1}\left\{(1-\beta )F\left\{x_{\mathrm{LR}-\mathrm{HOM}}\right\}\exp\left(-\frac{f^{2}}{2\sigma^{2}}\right)+\beta F\left\{x_{\mathrm{HR}-\mathrm{MAP}}\right\}\left(1-\exp\left(-\frac{f^{2}}{2\sigma^{2}}\right)\right)\right\},$$

where $F,F^{-1}$ denotes the Fourier transform operator and its inverse, respectively, and *f* is the spatial frequency, *σ* is the standard deviation of the Gaussian filter, and *β* is a weighting coefficient. Usually, we would set *β* to 0.85. We would typically use a standard incoherent apodizing function to shape the MAP-SIM spectrum before the final inverse FFT.

## 4. Results

**Figure 2:**
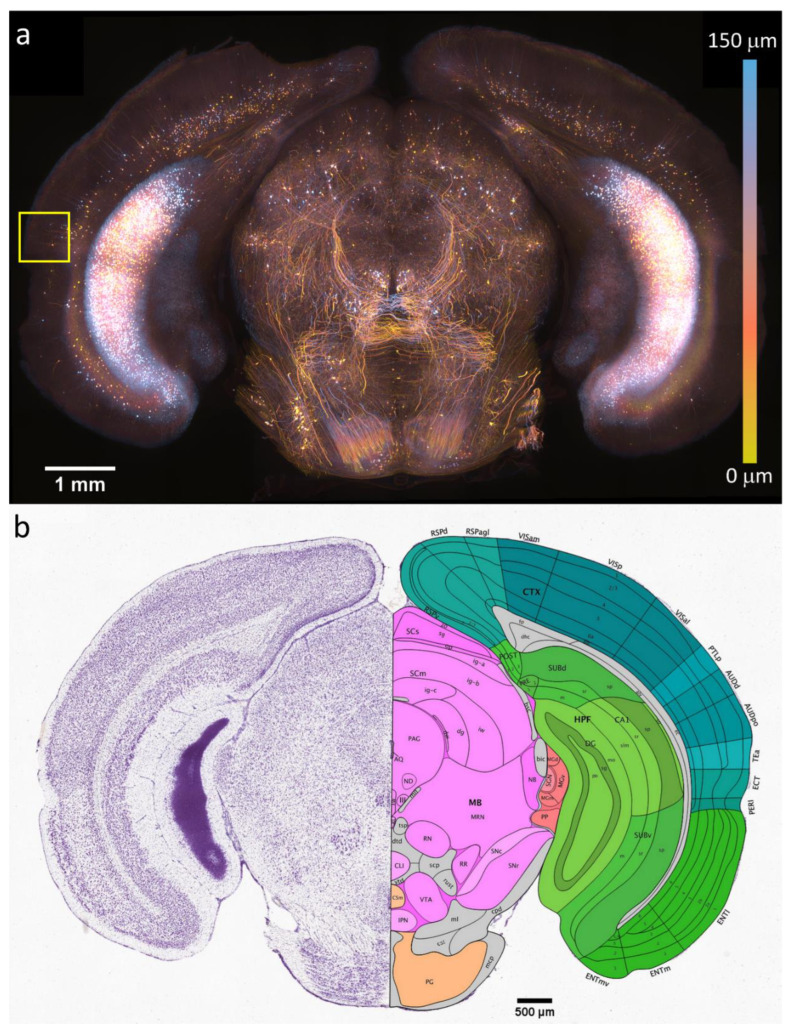
(**a**) Overview of the OS-SIM image. The yellow box indicates the temporal association area where the neurons were imaged with super-resolution MAP-SIM. (**b**) Nissl (**left**) and anatomical annotations (**right**) from the Allen mouse brain atlas and the Allen Reference Atlas—Mouse Brain, at the same slice position as (**a**) (slice 92 of 132, Allen Mouse Brain Atlas, mouse.brain-map.org and atlas.brain-map.org).

To acquire an overview of the slice with SIM methods, we first imaged using a 10×/0.4 NA water immersion objective. We acquired 60 image positions with a 20-percent overlap between each position and with 12 z-planes. In this image, the Z-plane spacing was 20 μm. Image stitching was accomplished using our lab’s methods and an ImageJ plugin [55], as shown in [46]. A composite image of the slice is shown in Figure 2, and it is color-coded based on depth using the isolum color table [56]. This image was acquired in 5 min and 30 s, with an additional 15 min and 35 s required for the OS-SIM processing, according to Equation (3). The final image was 8.4 GB in size, and it had 16,859 × 10,378 × 12 pixels.

**Figure 3:**
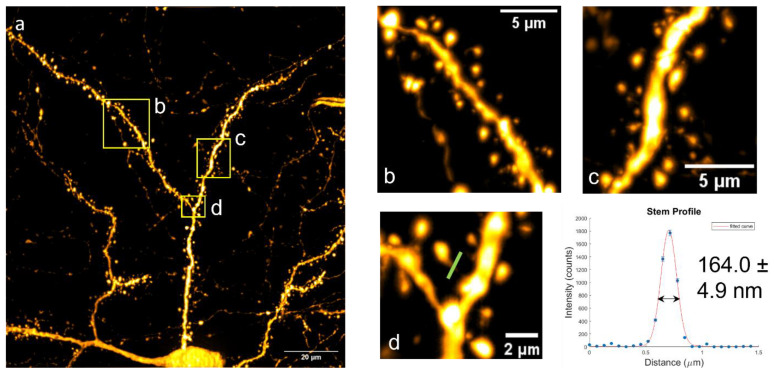
(**a**) TeA neuron imaged at a depth of 41 μm to 66 μm using a 100×/1.4 NA oil immersion objective. (**b**,**c**) Zoomed in views of the selected areas indicated in (**a**) by yellow boxes. The width of the spine neck, selected in (**d**), was fit to a Gaussian function (FWHM 164.0 ± 4.9 nm).

This slice was matched to Paxinos and Franklin’s mouse brain atlas [57] to identify which section of the brain was being imaged. Our slice was visually matched with slice 64. We further matched our sample to slice 92 of 132 in the Allen brain atlas [58,59]. Second order polynomial fits were made for both the horizontal and vertical directions using the edges and the central aqueduct as reference points. This allowed any point on this brain slice, recorded from the microscope stage coordinates, to be translated into the coordinates of the atlas. This method placed the neuron shown in Figure 3 in the temporal association area (TeA) of the mouse brain isocortex, as indicated by the yellow box in Figure 2a.

### Imaging Deep Neurons

To demonstrate MAP-SIM’s ability to image deeper into the sample than traditional SR-SIM, a TeA neuron 41–66 μm deep was imaged. The depth was measured using the closed-loop piezo stage. A 100×/1.4 NA oil immersion objective was used with an exposure time of 300 ms per SIM phase. This image is shown in Figure 3. The profile of a dendric spine neck was also measured (Figure 3d,e). The profile was fit in MatLab using a Gaussian function weighted by the square root of the counts, with nonlinear least squares methods. The full width at half-max (FWHM) was determined to be 164.0 ± 4.9 nm. To determine the image resolution, we calculated the power spectral density (PSD) as previously described [50]. We found that the WF image had a resolution of 247.6 nm while the MAP-SIM image had a resolution of 143.6 nm, an improvement of ~1.7-fold. These results are summarized in Table 1. Figures S1–S3 show additional images of cortical neurons imaged with MAP-SIM and a resolution analysis by a Fourier ring correlation (FRC) [60,61] and the PSD methods. The FRC measurements indicated a MAP-SIM resolution of approximately 150–160 nm, in good agreement with the 144 nm measured by the PSD methods.

**Figure 4:**
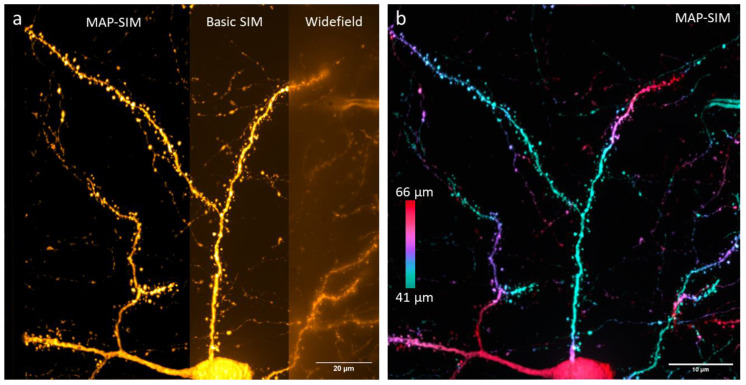
(**a**) TeA neuron shown in widefield, basic OS-SIM, and MAP-SIM. (**b**) MAP-SIM image color-coded by depth.

A comparison of widefield, basic OS-SIM (Equation (3)), and MAP-SIM (Equations (6) and (7)) for this same cortical neuron is shown in Figure 4 and further analyzed in Table 1. As is evident in the figure, widefield had the largest background due to out-of-focus light, with basic OS-SIM providing optical sectioning and MAP-SIM providing both optical sectioning and super-resolution. The imaging depth of 41–66 μm exceeded the depth limit of traditional SIM by approximately three-fold.

**Figure 5:**
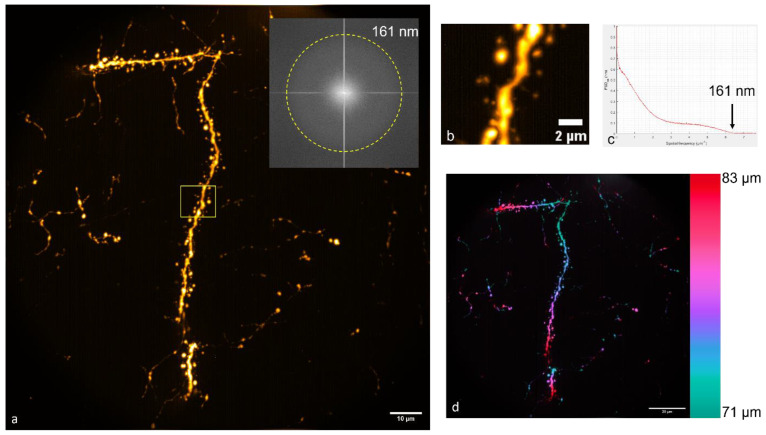
(**a**) TeA neuron imaged at a depth of 71 μm to 83 μm using a 100×/1.4 NA oil immersion objective. The inset shows the fast Fourier transform (FFT) of the image in (**a**), the boundary of which indicates the resolution. (**b**) Zoomed-in view of the selected area indicated in (**a**) by a yellow box. (**c**) A measurement of the resolution determined by measuring the power spectral density. (**d**) The MAP-SIM image color-coded by depth.

**Figure 6:**
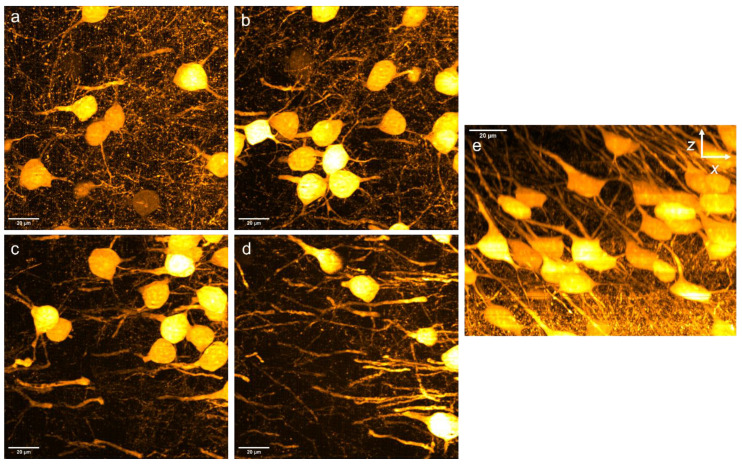
Neurons of the subiculum, ventral part, pyramidal layer (SUBv-sp), with an imaging depth of 0 to 113 μm (60×/1.42 NA oil immersion objective). The maximum-intensity projections of the imaged area have depths of (**a**) 0.2–28.4 μm, (**b**) 28.6–56.6 μm, (**c**) 56.8–84.8 μm, and (**d**) 85.0–113.0 μm. (**e**) X-Z projection of the imaged area.

We further imaged a neuron at a depth of 71–83 μm. This is shown in Figure 5. In this particular image, the resolution, measured by calculating the PSD, was 161 nm. While this was a decrease in resolution from the shallower neuron shown in Figure 3, it still surpassed the diffraction limit. Imaging at approximately 100 μm using these methods often resulted in images with large amounts of noise, and so the maximum imaging depth with a 100× objective in this sample appeared to be approximately 85 μm. Using a 60× objective, we were able to image up to 113 μm, as shown in Figure 6.

In addition to the cortical neurons imaged in Figure 3, Figure 4 and Figure 5 (and in Figures S1, S2 and S4), we also imaged an area of the brain in which a higher proportion of the neurons expressed the GFP marker (SUBv-sp subiculum, ventral part, pyramidal layer, also see Figure S5). This is shown in Figure 6. The maximum-intensity projection images, shown at different depths, showed good imaging at all depths. In addition, the imaging quality remained high even at depths past 100 μm using this objective (60× oil immersion).

**Figure 7:**
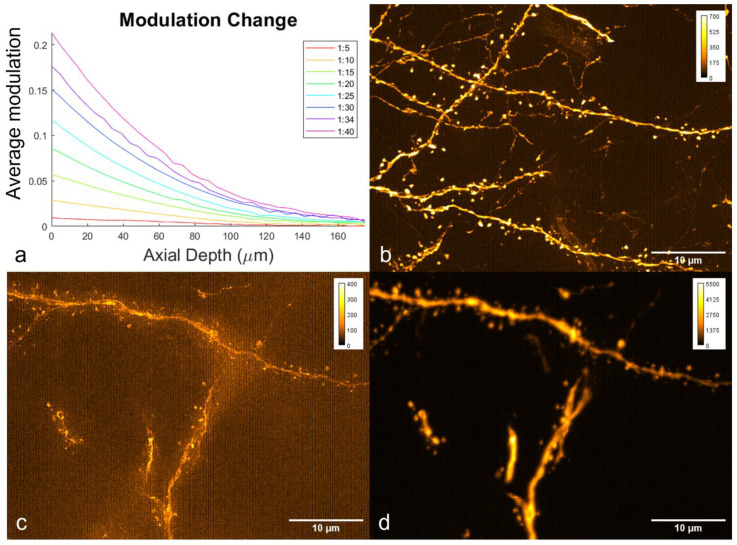
(**a**) Plot of modulation vs. axial depth for the different SIM patterns. (**b**) High-frequency pattern imaging of the TeA cortical neurons at the surface of the slice (0–10 μm). (**c**) High-frequency pattern imaging of the TeA cortical neurons at a depth of 41–45 μm. (**d**) Low-frequency pattern imaging of the same field of view shown in (**c**).

Typically, SIM uses high-frequency patterns to maximize the obtainable resolution, but it has limited imaging depth due to scattering and the generation of large amounts of background fluorescence. The pattern used here used a lower spatial frequency to penetrate deeper into the mouse brain while maintaining the pattern integrity. A comparison of (cropped) images acquired using a high-frequency pattern (i.e., one out of three microdisplay pixels was activated) and our lower-frequency pattern (i.e., two out of ten microdisplay pixels were activated) is shown in Figure 7. Also shown is a plot of the measured modulation of the SIM pattern vs. the depth for various SIM patterns using a thick fluorescent plastic slide (obtained from Chroma). The modulation, measured as the average of the (max − min/max + min) in a region of interest, fell as the pattern spatial frequency increased and as the depth increased. This was expected in the case of incoherent illumination, as we used here, because the incoherent optical transfer function applied [49]. The higher-frequency pattern resulted in a weaker signal and poorer image reconstruction when imaging deep into the sample, as shown in Figure 7c.

## 5. Discussion

By combining a structured illumination microscope with a large field-of-view and an image reconstruction method based on Bayesian statistics, we demonstrated synapse-resolving meso- and micro-scale volumetric imaging in an optically cleared coronal slice of adult mouse brain. The use of MAP-SIM and sample-optimized illumination patterns allowed us to collect super-resolution images well beyond the typical depth limit for SIM.

Compared to other super-resolution methods, SIM has poorer resolution. For example, the 144 nm lateral resolution achieved here is worse in comparison to the approximately 20 nm resolution that is typically achieved with STORM. However, SIM requires ~15 (or less, depending on the method used) images to reconstruct a super-resolution image. This is far lower than the 20,000 (or more) images usually required for STORM, making SIM imaging much faster and, therefore, a possibility for use when imaging live cells. The excitation power needed for SIM is much lower than that used in STORM. Here, we used 0.214 W/cm^2^ for SIM compared to the 2 kW/cm^2^ we previously used for STORM [62]. We found that the photobleaching in our experiments was minimal (Figure S6).

Most of the progress in super-resolution SIM has been in the acquisition and processing of images, but SIM was used in a detailed study on dendritic spines [9] where the authors developed a method for reconstructing and measuring the surface geometries of dendritic spines from 3D-SIM images. By adopting more flexible strategies for image acquisition and processing, such as the methods shown here, SIM is expected to be used more frequently in biological studies, including those on dense tissues such as brain tissue.

## Figures and Tables

**Table 1:** SNR and resolution measurements.

	SNR (dB)	Resolution (nm)
Widefield	43.87	247.6
Basic SIM	29.33	251.6
MAP-SIM	39.27	143.6

## Data Availability

The data are available upon request.

## Supplementary Materials

The following supporting information can be downloaded at: https://www.mdpi.com/article/10.3390/bioengineering10091081/s1, Table S1. Main components of the SIM system; Table S2. Camera parameters; Table S3. Light source parameters; Table S4. Microdisplay parameters; Table S5. Camera parameters for the machine vision camera used (only) in Figures S4 and S5; Table S6. Parameters of the imaging data; Figure S1: Cortical neuron; Figure S2: Enlarged views; Figure S3: Resolution analysis; Figure S4: Cortical neuron; Figure S5: Neurons of the midbrain; Figure S6: Photobleaching analysis; Figure S7: SIM system details; Figure S8: SIM system diagrams.
