# Supplementary material for: Super-Resolution Imaging of Neuronal Structures with Structured Illumination Microscopy
Source: Bioengineering (Basel). 2023 Sep 13;10(9):1081. doi: 10.3390/bioengineering10091081 (PMC10525192; doi:10.3390/bioengineering10091081)
Supplement: Supplementary file 1 [file bioengineering-10-01081-s001.zip › bioengineering-2617271-supplementary.pdf]

# Super-resolution imaging of neuronal structure with structured illumination microscopy – Supplementary information

**Tristan C. Paul, Karl A. Johnson, and Guy M. Hagen**

### Supplementary note 1: SIM system components, characteristics of the camera, microdisplay, and light source; parameters of the imaging data.

**Table S1.** Main components of the SIM system

| Component | Vendor | Vendor website | Part number |
|---|---|---|---|
| **Microdisplay** | Forth Dimension Displays | https://www.forthdd.com/ | SXGA-3DM |
| **Camera** | Andor | https://andor.oxinst.com/ | Zyla 4.2+ |
| **Light source** | Lumencor | https://lumencor.com/ | Spectra-X |
| **Microscope** | Olympus | https://www.olympus-lifescience.com/en/ | IX83 |
| **Stage** | Applied Scientific Instrumentation | https://www.asiimaging.com/ | PZ-2500FT and MS-2000-WK |
| **Mounting for microdisplay** | Thor Labs | https://www.thorlabs.com/ | PT3 |
| **Polarizing beam splitter** | Thor Labs | https://www.thorlabs.com/ | CCM1-PBS251 |
| **Optical lens (coupling from liquid light guide)** | Thor Labs | https://www.thorlabs.com/ | AC254-050-A-ML |
| **Optical lens (illumination tube lens)** | Olympus | https://www.olympus-lifescience.com/en/ | SWTLU-C |
| **Filters** | Chroma | https://www.chroma.com/ | 475/34 ex. filter T495lpxr dichroic ER525/50m em. filter |
| **Input/output card** | Measurement Computing | https://www.mccdaq.com/ | PCIM-DDA06/16 |

**Table S2.** Camera parameters

| Manufacturer | Andor |
|---|---|
| **Part number** | ZYLA-4.2P-CL10 |
| **Sensor type** | Front illuminated scientific CMOS |
| **Sensor size** | 13.3×13.3 mm |
| **Interface** | camera link |
| **Quantum efficiency** | 82% at 555 nm |
| **Read noise** | 0.9 $e^-$ |
| **Data range** | 16-bit |
| **Pixel size** | 6.5 µm |
| **Well depth** | 30,000 $e^-$ |
| **Resolution** | 2048×2048 pixels |
| **Maximum frame rate** | 100 FPS |
| **Readout method** | rolling shutter |

**Table S3.** Light source parameters

| Manufacturer | Lumencor |
|---|---|
| **Part number** | Spectra-X |
| **Output Wavelengths, nm** | 395, 440, 470, 550, 575, 640, 730 |
| **Turn-on time** | 10 µs |
| **Turn-off time** | 2 µs |

**Table S4.** Microdisplay parameters

| | |
|---|---|
| **Manufacturer** | Forth Dimension Displays |
| **Part number (system)** | SXGA-3DM |
| **Part number (microdisplay)** | M249 |
| **Part number (interface)** | R12 |
| **Microdisplay type** | Ferroelectric liquid crystal on silicon (FLCOS) |
| **Wavelength range** | 430-700 nm |
| **Resolution** | 1280×1024 pixels |
| **Switching speed** | 40 µs |
| **Active area** | 17.43 mm×13.95 mm<br>22.3 mm diagonal |
| **Pixel size** | 13.62 µm |
| **Inter pixel gap** | 0.54 µm |
| **Maximum frame rate** | 3.2 KHz binary images |
| **Fill factor** | 93% |
| **Reflectance in 'ON' state** | 60% |
| **Onboard memory** | 768 binary images |

**Table S5.** Camera parameters for the machine vision camera used (only) in Figs. S4 and S5.

| | |
|---|---|
| **Manufacturer** | Teledyne FLIR |
| **Part number** | BFS-U3-244S8M-C |
| **Camera model** | FLIR blackfly S |
| **Sensor type** | Back illuminated CMOS |
| **Sensor type** | Sony IMX540 |
| **Sensor size** | 19.36 mm diag. |
| **Interface** | USB3 |
| **Quantum efficiency** | 69% at 525 nm |
| **Read noise** | 2.31 $e^-$ |
| **Data range** | 12-bit |
| **Pixel size** | 2.74 µm |
| **Well depth** | 9648 $e^-$ |
| **Resolution** | 5320×4600 pixels |
| **Maximum frame rate** | 15 FPS |
| **Readout method** | global shutter |

**Table S6.** Parameters of the imaging data

| | **Camera** | **Objective (Olympus)** | **Exposure time** | **SIM pattern*** | **Z-spacing and num. slices** | **Acquisition time Processing time** |
|---|---|---|---|---|---|---|
| **Fig. 2** | Andor Zyla | UPLAPO 10×/0.4 NA water immersion | 100 ms | 2-10-1-12 | 12 slices Spacing 20 um (10x6 pano.) | 5:30 to aquire 15:35 proc. time |
| **Fig. 3&4** | Andor Zyla | UPLSAPO 100×/1.4 NA oil immersion | 300 ms | 2-10-1-12 | 126 slices Spacing 200 nm | 19 min to acquire 36 min proc. time |
| **Fig. 5** | Andor Zyla | UPLSAPO 100×/1.4 NA oil immersion | 800 ms | 2-10-1-12 | 61 slices 200 nm spacing | 22 min to acquire 17 min proc. time |
| **Fig. 6** | Andor Zyla | UPLXAPO 60×/1.42 NA oil immersion | 100 ms (40% power) | 2-10-1-12 | 566 slices 200 nm spacing | 62 min to acquire 73 min proc. time |
| **Fig. 7a** | Andor Zyla | UPLSAPO 20×/0.75 NA air | 150 ms | Various | 35 slices 5 um spacing | Various |
| **Fig. 7b** | Andor Zyla | UPLSAPO 100×/1.4 NA oil immersion | 250 ms | 1-2-1-3 | 51slices 200 nm spacing | 2 min to acquire 3 min proc. time |
| **Fig. 7c** | Andor Zyla | UPLSAPO 100×/1.4 NA oil immersion | 250 ms | 1-2-1-3 | 21 slices 200 nm spacing | 2 min to acquire 1 min proc. time |
| **Fig. 7d** | Andor Zyla | UPLSAPO 100×/1.4 NA oil immersion | 250 ms | 2-10-1-12 | 21 slices 200 nm spacing | 5 min to acquire 4 min proc. time |
| **Fig. S1&S2** | Andor Zyla | UPLSAPO 100×/1.4 NA oil immersion | 400 ms | 1-9-1-10 | 47 slices 200 nm spacing | 26 min to acquire 17 min proc. time |
| **Fig. S4** | FLIR Blackfly S | UPLSAPO 60×/1.35 NA oil immersion | ND** | 1-9-1-10 | 69 slices 145 nm spacing | ND** 17 min proc. time |
| **Fig. S5** | FLIR Blackfly S | UPLAPO 10×/0.4 NA Oil immersion | ND** | 1-10-1-11 | 9 slices 15 um spacing | ND** 11 min proc. time |
| **Fig. S6** | Andor Zyla | UPLXAPO 60×/1.42 NA oil immersion | 100 ms (20% power) | 2-10-1-12 | No Z in this experiment | 30 min to acquire 6 min proc. time |

* A SIM pattern of 2-10-1-12 denotes 2 columns of pixels ON, 10 columns of pixels OFF, an inter-pattern shift of 1 pixel, and a total of 12 patterns in the set. Similarly pattern 1-9-1-10 denotes 1 column of pixels ON, 9 columns of pixels OFF, a shift of 1 pixel and a total of 10 patterns in the set.

** Some of the metadata was lost for these experiments; the exposure time was ~200 ms. Acquisition times were about 10-15 minutes.

**Supplementary note 2: supplementary figures showing additional images of cortical neurons and resolution analysis by Fourier ring correlation (FRC) and power spectral density (PSD) methods.**

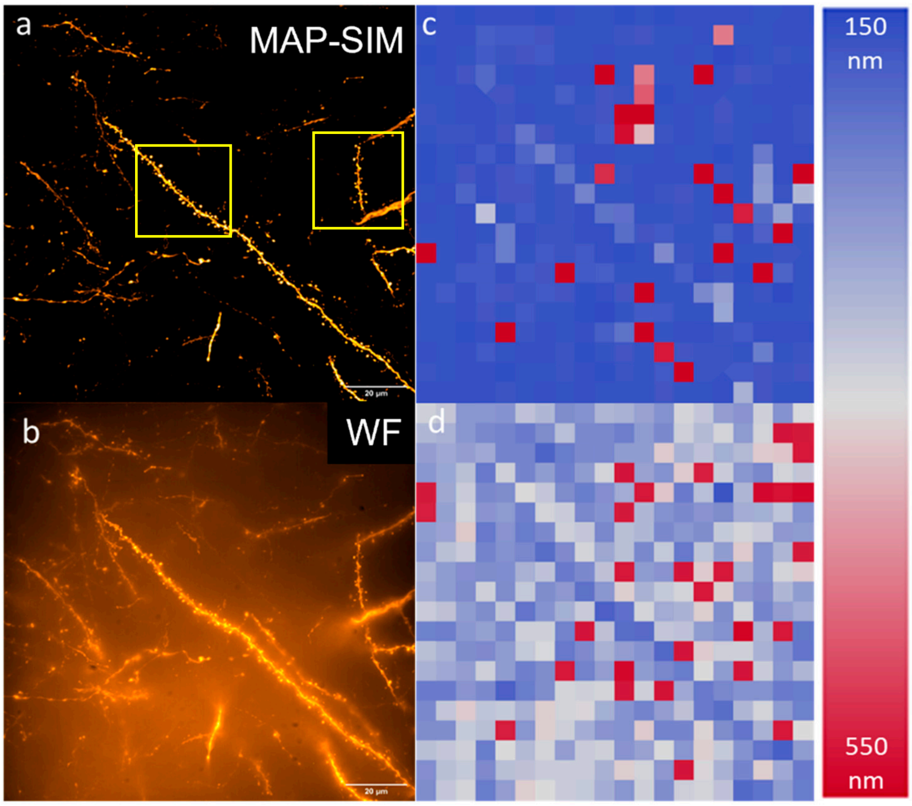


Figure S1: Cortical neuron. Imaged with (a) MAP-SIM, (b) conventional widefield, (c) FRC measurement of MAP-SIM, (d) FRC measurement of WF. FRC measurements [60] were performed using NanoJ-squirrel [61].

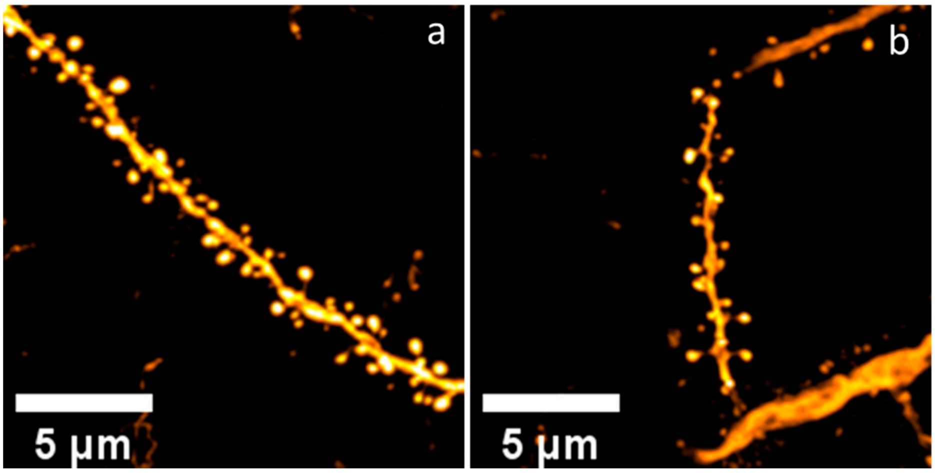


Figure S2: Enlarged views. (a, b) Areas indicated by yellow boxes in Figure S1a.

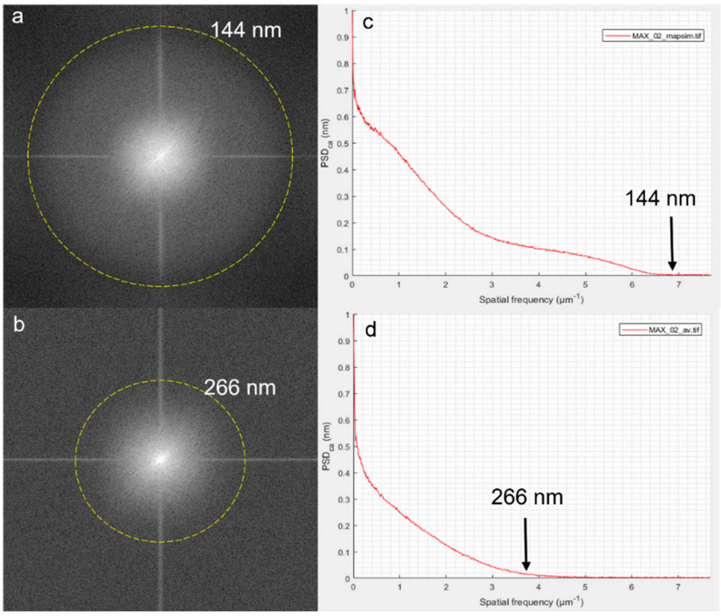


Figure S3: Resolution analysis. (a, c) FFT of Figure S1a and corresponding power spectral density measurement, (b,d) FFT of Figure S1b and corresponding power spectral density (PSD) measurement. PSD was measured using SRMeasureToolbox[50].

**Supplementary note 3: supplementary figures showing images of cortical neurons and neurons of the midbrain imaged with a machine vision camera**.

Figures S4 and S5 show SIM images of a cortical neuron, and neurons of the midbrain, acquired with a machine vision camera at two different magnifications. This 24.5 megapixel camera is low cost compared to the Andor Zyla camera, but has adequate performance for this application. Table S5 shows parameters of this particular camera. This data illustrates how the SIM system described here can be adapted for use with lower cost components, making this system more accessible to a larger number of investigators. The complete SIM system described here is much lower in cost than other optical sectioning microscopes such as point scanning confocal or spinning disk systems, while also offering super-resolution.

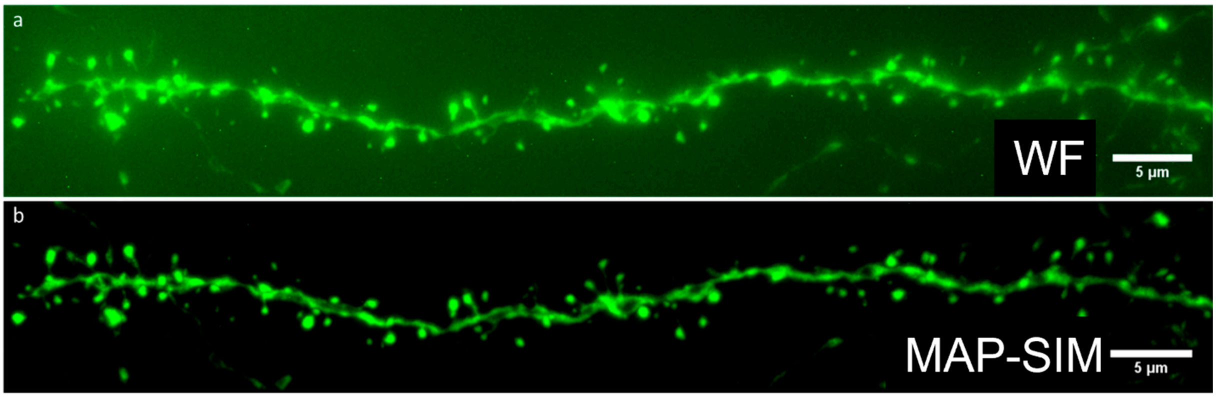


Figure S4: Cortical Neuron. Imaged with (a) conventional widefield and (b) MAP-SIM. Objective: Olympus UPLSAPO 60×/1.35 NA oil immersion. Camera: FLIR Blackfly S.

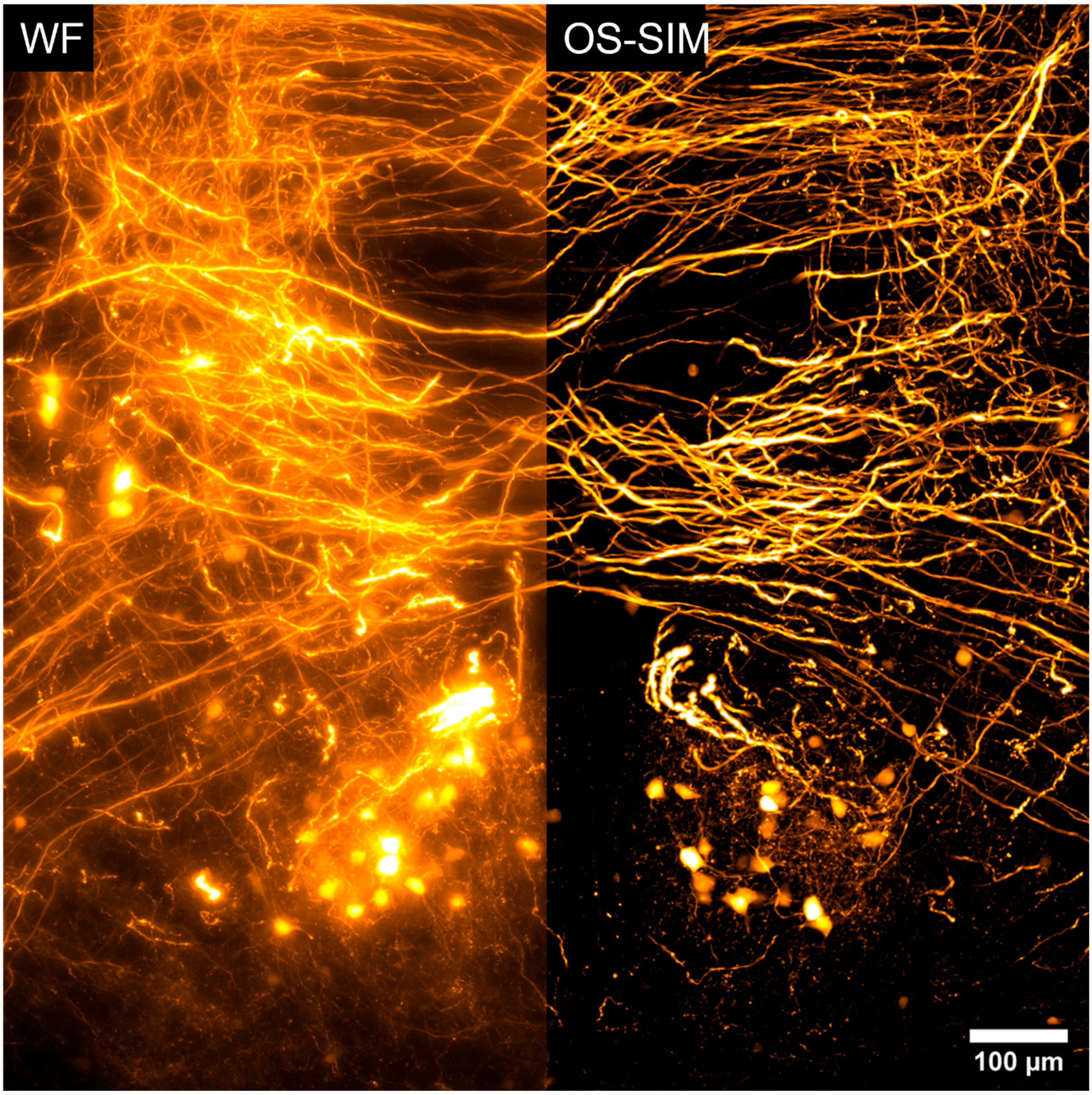


Figure S5: Neurons of the midbrain. Imaged with (left) WF, and (right) OS-SIM. Objective: Olympus UPLANAPO 10×/0.4 NA oil immersion. Camera: FLIR Blackfly S.

**Supplementary note 4: supplementary figure showing analysis of photobleaching during SIM imaging.**

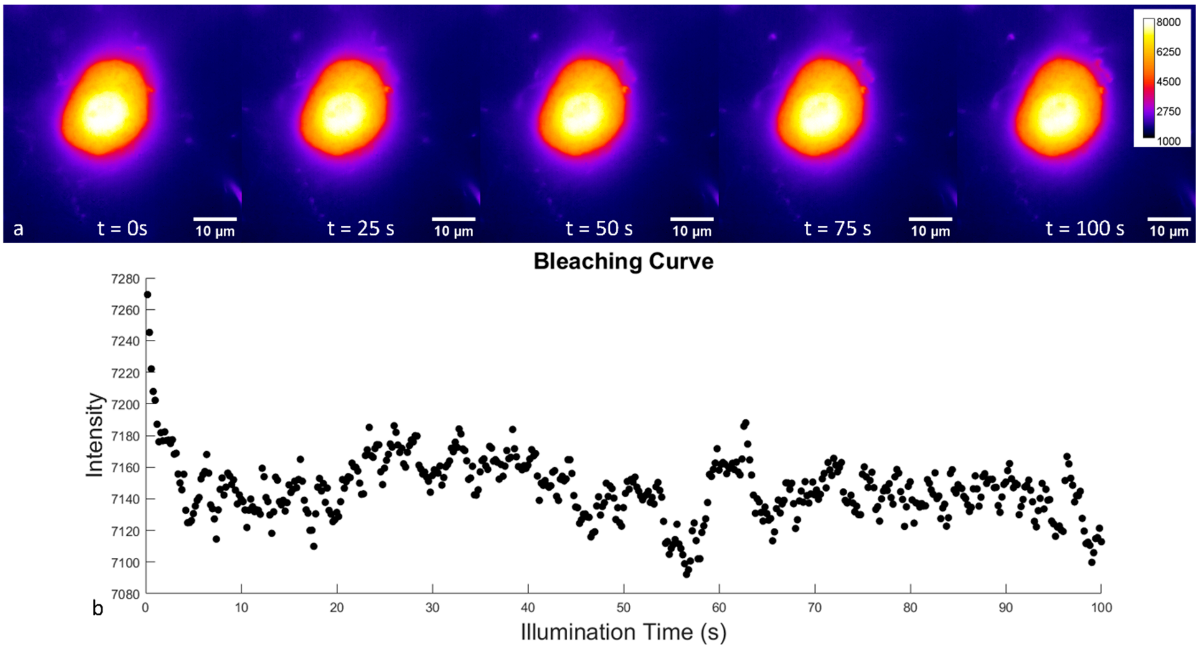


Figure S6: Photobleaching analysis. (a) Images of a neuron over time during continuous exposure using the 2-10-1-12 pattern. Images were taken using a 60× objective with an exposure time of 100 ms. (b) Average intensity over time of the boxed area in Figure S6a. Intensity decreased only 1.7% after 100 s of illumination.

**Supplementary note 5: additional details about the setup used in SIM imaging.**

While the camera and XYZ stage receive signals directly from Andor IQ software and an input/output computer card (Measurement Computing DDA06/16), the illumination signals which the software generates must be altered before being sent to the light source. Firstly, the microdisplay used in our setup will not produce an image on the sample if it is illuminated with a constant light source. Rather, a meaningful illumination pattern will only form if the light source is synchronized with an enable signal output from the microdisplay control board. Therefore, the channel selection signals output by IQ software and the I/O card are first modulated with the microdisplay enable signal. This is performed by the leftmost AND gates pictured in Figure S7 (see also Figure 1 of the main text). Additionally, to reduce unnecessary light exposure to the sample, the light source is shut off whenever the camera sensor is not being exposed. This is accomplished by performing a second logical AND of the result of the previous AND with the 'FIRE' signal output from the camera. This process is illustrated in Figure S7.

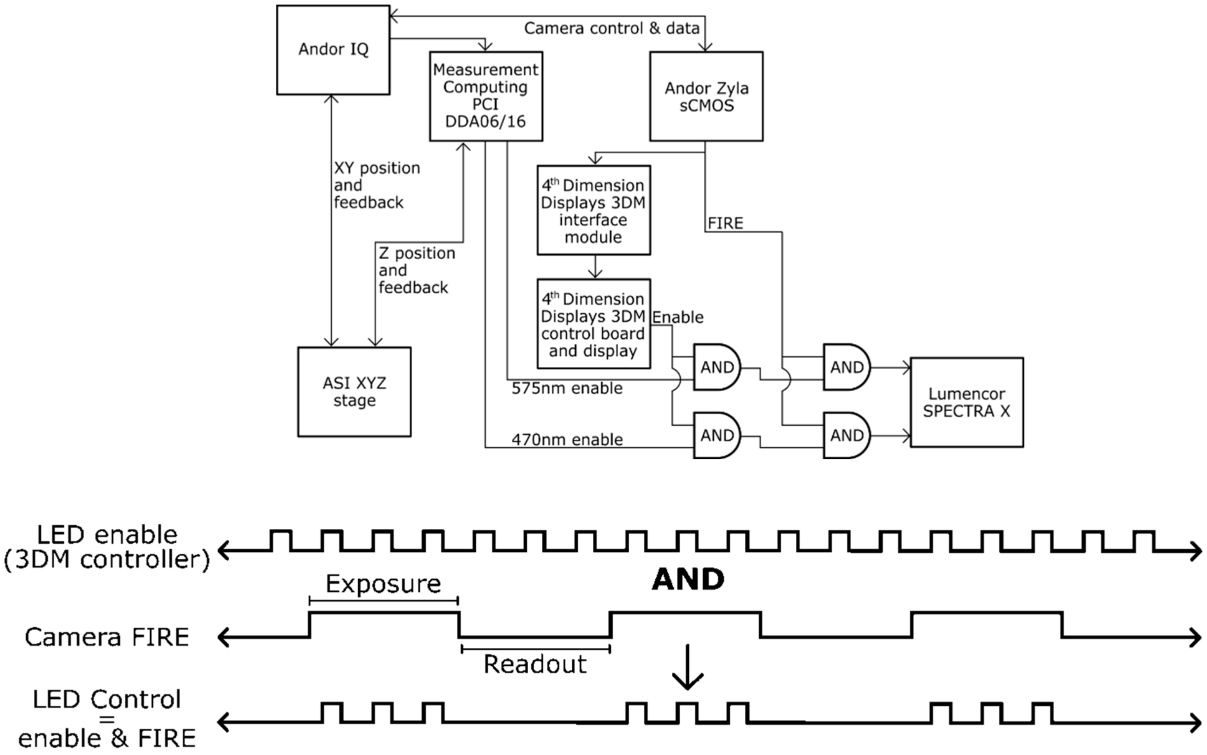


Figure S7: SIM system details. (above) connection diagram (below) schematic of the timing scheme.

Figure S8a shows a diagram of the optical system. Figure S8b shows the principle of the microdisplay operation. The microdisplay is a ferroelectric, liquid crystal-on-silicon device which functions as an array of quarter wave plates with a reflective backing. Each pixel of the array can be individually turned ON or OFF. In the ON state, the quarter wave plate in that pixel converts linearly polarized light to circular polarization. Vertically polarized light reflects off of a polarizing beam splitter cube and into the device where it passes through the quarter wave plate, reflects off the mirror-like backing, and passes a second time through the quarter wave plate. This has the effect of converting vertically polarized light to horizontally polarized light, which then passes through the polarizing beam splitter cube and enters the microscope. Use of an epifluorescence microscope with filter cubes in a rotating turret lets one easily perform multi-color imaging. Multi-band filter sets and electronically controlled light sources allow multi-color structured illumination microscopy with no moving parts. 3D imaging is accomplished slice-by-slice by changing the sample focus. We have successfully used microscopes from Olympus (IX71, IX83) and Leica (DMI8) in similar SIM systems.

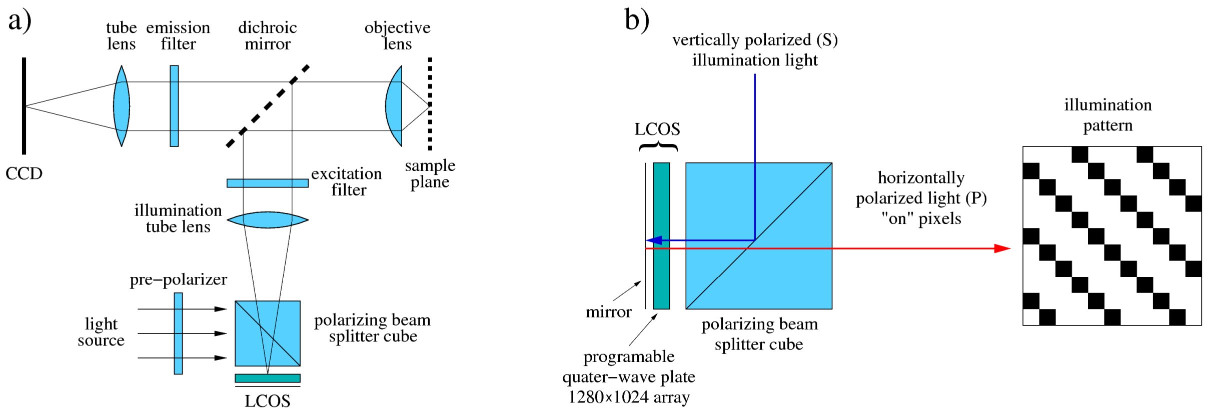


Figure S8: SIM system diagrams. (a) simplified diagram of the system (b) illustration of microdisplay operation.

**Supplementary note 6: SIMToolbox resources.**

SimToolbox homepage: https://github.com/simtoolbox
SIMToolbox[45] was used to process all of the data in this paper. SIMToolbox is an open-source, modular set of functions for MATLAB equipped with a user-friendly graphical interface and designed for processing two-dimensional and three-dimensional data acquired by structured illumination microscopy. Both optical sectioning and super-resolution applications are supported. The software is also capable of maximum a posteriori probability image estimation (MAP-SIM), the method used in this paper.

Test files: https://zenodo.org/record/4044159#.YOYRe35MEuU
This site contains data for testing SIMToolbox and for comparison with data acquired with other SIM systems. The available data includes images acquired with our SIM system, and also data acquired with Zeiss and Nikon SIM systems.

SIMToolbox documentation: https://github.com/simtoolbox/Documentation
This site contains documentation related to SIMToolbox, including quick-start guides for processing data acquired with our system or with Nikon systems.

**Supplementary references**

60. Nieuwenhuizen, R.P.J.; Lidke, K. a; Bates, M.; Puig, D.L.; Grünwald, D.; Stallinga, S.; Rieger, B. Measuring image resolution in optical nanoscopy. *Nat. Methods* **2013**, *10*.
61. Culley, S.; Albrecht, D.; Jacobs, C.; Pereira, P.M.; Leterrier, C.; Mercer, J.; Henriques, R. Quantitative mapping and minimization of super-resolution optical imaging artifacts. *Nat. Methods* **2018**, *15*, 263–266.
50. Pospíšil, J.; Fliegel, K.; Klíma, M. Assessing resolution in live cell structured illumination microscopy. In Proceedings of the Proceedings of SPIE - The International Society for Optical Engineering; Páta, P., Fliegel, K., Eds.; SPIE, 2017; Vol. 10603, p. 39.
45. Křížek, P.; Lukeš, T.; Ovesný, M.; Fliegel, K.; Hagen, G.M. SIMToolbox: A MATLAB toolbox for structured illumination fluorescence microscopy. *Bioinformatics* **2015**, *32*, 318–320.
